# Comparison of mid-term outcomes between unilateral biportal endoscopic and minimally invasive transforaminal lumbar interbody fusion in the treatment of single-level lumbar degenerative disease

**DOI:** 10.1371/journal.pone.0321569

**Published:** 2025-04-29

**Authors:** Xuelei Zhang, Qiumei Yuan, Yu Zhang, Zuchao Gu, Guo Li

**Affiliations:** 1 Department of Orthopedics, Chengdu Integrated TCM & Western Medicine Hospital/Chengdu First People’s Hospital, Chengdu, Sichuan Province, China; 2 Department of Anesthesia and Surgery Center, Chengdu Integrated TCM & Western Medicine Hospital/Chengdu First People’s Hospital, Chengdu, Sichuan Province, China; Mie University Graduate School of Medicine, JAPAN

## Abstract

**Objective:**

To compare the mid-term clinical and radiological outcomes between unilateral biportal endoscopic transforaminal lumbar interbody fusion (ULIF) and minimally invasive transforaminal lumbar interbody fusion (MIS-TLIF) in the treatment of single-segment lumbar degenerative disease.

**Methods:**

Patients with L4–S1 disease treated with fusion surgery in our department between August 1, 2019 and June 30, 2020 were retrospectively analyzed. The patients were categorized into ULIF and MIS-TLIF groups based on the surgical method performed. The preoperative demographic baseline and operation-related indicators of the groups were compared, including operative time, estimated blood loss (EBL), postoperative drainage volume, time to ambulation, and postoperative hospital stay. The Visual Analog Scale (VAS) was utilized to assess the severity of back pain (VAS-B) and leg pain (VAS-L). The Oswestry Disability Index (ODI) and Japanese Orthopedic Association (JOA) scores were employed to evaluate the level of functionality. Bridwell criteria were used to evaluate interbody fusion. The lumbar lordotic angle (LLA), intervertebral disc height (IDH), and segmental lordotic angle (SLA) pre- and post-operatively were compared. The creatine kinase (CK), C-reactive protein (CRP), erythrocyte sedimentation rate (ESR), and interleukin-6 (IL-6) levels pre- and post-operatively, and the complication rates were compared.

**Results:**

The baseline preoperative demographics of the ULIF (n=35) and MIS-TLIF (n=42) groups did not differ significantly. Compared with MIS-TLIF, ULIF had lower intraoperative blood loss and postoperative drainage volume and shorter time to ambulation and postoperative hospital stay, but longer operative time. The VAS-B, VAS-L, JOA, and ODI scores of both groups significantly improved. The VAS-L at 1 week postoperatively, the VAS-B at 1 week and 1 month postoperatively, and the JOA and ODI scores at 1 month postoperatively were better in the ULIF group. At 1 and 3 days postoperatively, the ULIF group exhibited substantially reduced levels of CRP, CK, and IL-6. The fusion rates did not differ significantly at 1 year, 2 years, and 3 years of follow-up. The IDH, SLA, and LLA improved significantly in both groups but no significant differences were observed between the two groups. Complication rates were comparable between the two groups.

**Conclusions:**

Both ULIF and MIS-TLIF are proven to be safe and effective minimally invasive lumbar fusion techniques. Both achieve comparable outcomes in terms of interbody fusion rate, long-term pain relief, functional improvement, and complication rate. Compared with MIS-TLIF, ULIF has less intraoperative blood loss, less postoperative drainage volume, reduced inflammatory reaction, and faster postoperative pain relief and functional improvement.

## Introduction

Lumbar degeneration disease (LDD), such as lumbar disc herniation, lumbar spondylolisthesis, and lumbar spinal stenosis, is caused by degenerative changes in lumbar intervertebral discs, lumbar facet joints, and ligaments. The main clinical manifestations are back pain and radiating lower limb pain or numbness. Lumbar degenerative disease is among the most common and expensive disabling diseases in middle-aged and elderly people. Its annual prevalence is increasing with aging population [1,2]. In cases with conservative treatment failure, lumbar fusion surgery is the main choice for segment decompression, reduction, fusion, and stabilization.

Open posterior lumbar interbody fusion, including transforaminal and posterior lumbar interbody fusion (TLIF and PLIF, respectively) is widely used to treat LDD [3,4]. However, extensive dissection of paraspinal muscles in open surgery increases intraoperative blood loss and postoperative muscle atrophy, which affect surgical outcomes and recovery [5,6]. Hence, minimally invasive surgical methods are needed.

Foley et al. first introduced the concept of MIS-TLIF in 2003 [7]. Compared with PLIF or TLIF, MIS-TLIF provides reduced soft tissue injury, estimated blood loss (EBL), and postoperative pain; faster postoperative recovery; lower complication rate; and shorter postoperative hospital stay [8,9]. However, MIS-TLIF is performed in the working channel, with narrow surgical space and limited intraoperative visual field, which may lead to insufficient nerve decompression and, ultimately, reduced surgical outcomes [10,11]. Thus, techniques that simultaneously ensure sufficient surgical field of view, wide operating space, and reduced surgical damage are needed. In 2017, Heo et al. reported good short-term clinical and radiological results in patients with LDD who treated with ULIF [12]. Recent studies have reported less EBL and quicker recovery for ULIF compared with MIS-TLIF for LDD [13,14]. As per the literature’s definition of follow-up duration for lumbar fusion surgery, a follow-up period of 20 months or less is considered short-term, while a period more than 34.5 months is considered long-term [15], the median follow-up times of available reports on ULIF (6–18 months) are insufficient to verify the medium-to-long-term safety and effectiveness [16].

Therefore, this study compared the mid-term clinical efficacies between ULIF and MIS-TLIF in treating single-segment LDD, with a median follow-up time of more than 40 months.

## Materials and methods

### Study design and participants

This retrospective cohort study included patients with L4–S1 single-segment LDD treated with fusion surgery in our hospital between August 1, 2019 and June 30, 2020. Two senior spine surgeons performed ULIF and MIS-TLIF, respectively. The patients were included in the ULIF or MIS-TLIF group depending on the surgical approach according to the surgeon’s preference. This study followed the principles of the Declaration of Helsinki and its 2013 revision. The protocol received approval from the Ethics Committee of Chengdu First People’s Hospital (identification code 2023 ZXKT No. 016), and the requirement for informed patient consent was waived due to the retrospective study design. The data were accessed for research purposes from October 5, 2023, to December 10, 2023. The authors had no access to information that could identify individual participants during or after data collection.

Inclusion criteria: (1) lumbar spinal stenosis, grade I–II lumbar spondylolisthesis (isthmic or degenerative) diagnosis; (2) single-level fusion; (3) age ≥40 years; (4) follow-up time ≥3 years; and (5) lower lumbar fusion (L4–S1). Exclusion criteria: (1) previous lumbar fusion; (2) lumbar fracture, tumor, or infection; (3) imaging-indicated congenital spinal hypoplasia or deformity; (4) severe scoliosis (Cobb angle >30°); and (5) cardiopulmonary insufficiency or inability to tolerate general anesthesia.

### Surgical procedures

**Fig 1:**
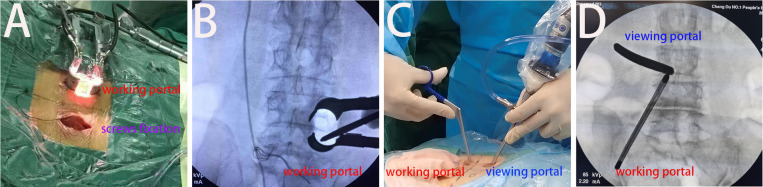
Intraoperative photographs and radiographs of both procedures. A-B show the working channel in MIS-TLIF group; C-D show the working channel in ULIF group.

For MIS-TLIF procedure, the patient was positioned face down on a fluoroscopic bed after general anesthesia. The surgical segment and incision were located under C-arm fluoroscopy and marked. A 4-cm longitudinal incision was made on the affected side 3 cm beside the spinous process and the soft tissues were incised sequentially.The working channel and optical fiber were placed along the gap between the longissimus and multifidus muscles to expose the articular processes of the target segment (Fig 1A–1B). The inferior and part of the superior articular process were resected, the nerve root canal and lateral recess were enlarged, and dural sac and nerve root compression was relieved. For patients with bilateral symptoms, the operating table was tilted 15–20° opposite to the doctor’s position, and the working channel was adjusted to expose the inferior portion of the superior spinous process. The contralateral ligamentum flavum and the inferior part of the lamina were resected. The contralateral lateral recess was decompressed, and the traveling nerve root was exposed. The space between the disc and the ligamentum flavum, the nerve roots in the pedicle area, and the exit area were thoroughly explored and decompressed. Preparation of the bone graft bed in the intervertebral space. Normal saline was used to thoroughly wash the incision and completely stop bleeding. Under C-arm fluoroscopy, autologous bone and a PEEK cage were sequentially implanted for interbody fusion. The spinal and bilateral nerve root canals and lateral recess were re-explored to confirm adequate decompression and relief of nerve root and dural sac compression. Four percutaneous pedicle screws and two connecting rods were inserted under C-arm fluoroscopy, and the tail cap was fixed. The incision was irrigated, and the bleeding was stopped thoroughly. A negative pressure drainage tube was inserted into the operative area, and the incision was closed sequentially.

For ULIF, the patients were placed prone on an X-ray fluoroscopy bed after general anesthesia. The surgical segment and incision were located under C-arm fluoroscopy and marked. The incision was performed along the medial border of the two pedicles on the operative side on anteroposterior radiographs and 1 cm above and below the middle of the intervertebral space on lateral radiographs. Then, the surgical site was thoroughly cleansed and covered with sterile and waterproof surgical drapes. Two 1-cm surgical incisions were made according to the skin marks, and the skin and fascia were incised successively (if the left side was the surgical side, cephalic and caudal incisions were made for the endoscopic and working channels, respectively). The soft tissue channels were expanded using a serial dilator. The stripper was employed to remove the soft tissue off the surface of the lamina. The bipolar radiofrequency device was placed in the working channel, while the endoscope and irrigation system were placed in the endoscopic channel (Fig 1C–1D). Hemostasis and further removal of residual soft tissue on the lamina surface were performed. An electric grinding drill and rongeur were used to remove part of the lamina, facet joint, and all ligamentum flavum on the ipsilateral side to expose and decompress the central canal and nerve roots on that side. If the patient had bilateral symptoms, the contralateral side was decompressed by removing part of the lamina, facet joint, and all ligamentum flavum through the space between the lamina and dural sac. Under imaging monitoring, the disc and cartilage endplate were removed using the transforaminal approach. Prior to the insertion of a cage, substantial quantities of fusion materials, including autologous bone chips and hydroxyapatite particles containing bone morphogenetic proteins (BMPs), were introduced into the disc space with a customized funnel. A drainage tube was placed. The surgical incision was sutured layer by layer. A sterile dressing was used to cover the surgical incision.

### Postoperative management

For both procedures, antibiotics were administered for 24 h postoperatively to prevent wound infection. The criterion for drain removal was <30 mL of drainage in the previous 24 h. The patients were instructed to wear a spinal brace for ambulation after drain removal.

### Variables and measurements

The baseline demographic information included sex, age, body mass index (BMI), diagnosis, disease duration, surgical segment, and presence of diabetes and hypertension. The perioperative data included operative time, EBL, postoperative drainage volume, postoperative time to ambulation, perioperative complications, and postoperative hospital stay. The data on inflammatory factors included ESR and CRP, CK, and IL-6 concentrations preoperatively and 1, 3, and 7 days postoperatively (For patients with a postoperative hospital stay of <7 days, tests were performed in the outpatient clinic). VAS scores were used to evaluate VAS-B and VAS-L pain 2 days preoperatively and 1 week, 1 month, 3 months, 12 months, and 36 months after the surgery. The ODI and JOA scores were used to assess overall function at 2 days preoperatively and 1 month, 6 months, 12 months, 24 months, and 36 months postoperatively. IDH, SLA, and LLA were evaluated on anteroposterior and lateral X-ray films preoperatively and 3 months, 12 months, 24 months, and 36 months postoperatively. The IDH was measured by Hurxthal-II method. The SLA and LLA were assessed via the Cobb method [17,18]. Bridwell criteria were used to judge interbody fusion grade at 12 months, 24 months, and 36 months postoperatively. Grade I or II fusion was considered clinical fusion [19].

### Statistical analysis

All statistical analyses were performed using IBM SPSS Statistics for Mac, version 27.0 (IBM Corp., Armonk, NY, USA). Continuous variables with normal distributions are expressed as means ± standard deviations. The independent t-test was used to compare differences between groups. One-way repeated-measures analysis of variance was used to compare data at different times within groups. Data with non-normal distributions were expressed as medians (first and third quartiles), and inter-group differences were examined using the Mann–Whitney U test. Categorical variables are expressed as frequencies or percentages and inter-group differences were assessed using chi-square or Fisher’s exact tests. *P*<0.05 was considered statistically significant.

## Results

The demographic data are shown in Table 1. Out of the total of 77 patients, 35 were included in the ULIF group and 42 were recruited in the MIS-TLIF group. There were no significant differences between the two groups in terms of sex composition, average age, BMI, diagnosis, surgery segment, disease duration, prevalence of diabetes and hypertension, and follow-up time (*P*>0.05).

**Table 1:** Demographics of the study sample.

	ULIF	MIS-TLIF	*P* value, t/U/χ^2^ value
No. of patients	35	42	–
Sex (M/F)	14/21	18/24	*P*=0.821, χ^2^=0.064
Mean age (year)	64.1±8.5	62.3±6.4	*P*=0.286, t=1.076
BMI, kg/m^2^	25.5±3.7	25.2±3.5	*P*=0.687, t=0.404
Preoperative diagnosis			*P*=0.794, χ^2^=0.462
Stenosis	27	34	
Spondylolisthesis	6	5	
Recurrent disc herniation	2	3	
Operation segment			*P* =1.000, χ^2^=0.068
L4-5	25	31	
L5-S1	10	11	
Hypertension	11	15	*P*=0.833, χ^2^=0.045
Type II diabetes mellitus	8	9	*P*=0.880, χ^2^=0.023
Disease duration (month)	25.3±6.9	23.5±6.3	*P*=0.233, U=618.5
Follow up (month)	41.2±3.5	42.5±4.1	*P*=0.145, t=-1.473

ULIF, unilateral biportal endoscopic lumbar interbody fusion; MIS-LIF, minimally invasive transforaminal lumbar interbody fusion; BMI, body mass index; Boldface indicates statistical significance; Data presented as mean ± SD.

The perioperative information is detailed in Table 2. Significantly prolonged was the mean operative time in the ULIF group (*P*<0.001) than in the MIS-TLIF group. Significantly less mean EBL occurred in the ULIF group compared to the MIS-TLIF group (*P*=0.018). The mean postoperative drainage volume in the ULIF group was considerably lower than that in the MIS-TLIF group (*P*<0.001). In contrast to the MIS-TLIF group, the ULIF group exhibited a considerably reduced mean time to ambulation following surgery (*P*<0.001). The mean postoperative hospital stay also differed significantly between the MIS-TLIF and ULIF groups (*P*=0.004).

**Table 2:** Surgery-related information for the ULIF and MIS-TLIF groups.

	ULIF	MIS-TLIF	*P* value, t value
Operative time (minute)	176.5±27.7	149.3±20.9	***P<*0.001, t=4.917**
Estimate blood loss (ml)	187.9±32.6	205.5±31.0	***P*=0.018, t=-2.420**
Drainage volume (ml)	76.1±21.1	95.7±18.4	***P<*0.001, t=-4.378**
Time to ambulation (hour)	28.1±7.4	35.3±7.6	***P<*0.001, t=-4.142**
Postop hospital stay (day)	8.1±3.2	10.5±3.8	***P*=0.004, t=-2.934**

Boldface indicates statistical significance; Data presented as mean ± SD.

The inflammation factors are shown in Table 3. There was no significant difference in the mean preoperative levels of CRP, CK, ESR, and IL-6 between the ULIF and MIS-TLIF groups (*P*>0.05). The mean levels of CRP, CK, ESR, and IL-6 in both groups were considerably elevated on days 1 and 3 after surgery compared to before surgery (all *P*<0.05).

**Table 3:** Preoperative and postoperative values of CRP, CK, ESR and IL-6.

Variables	ULIF	MIS-TLIF	*P* value, t value
CRP (mg/L)			
Preop	4.7±2.3	4.5±2.4	*P*=0.724, t=0.354
1 day postop	16.5±3.0^*^	18.6±2.8^*^	***P*=0.002, t=-3.254**
3 days postop	24.5±2.7^*^	27.3±2.6^*^	***P<*0.001, t=-4.661**
7 days postop	12.4±2.5^*^	12.8±2.2^*^	*P*=0.481, t=-0.709
CK (U/L)			
Preop	86.1±37.5	83.2±34.9	*P*=0.729, t=0.348
1 day postop	283.5±57.3^*^	335.2±53.4^*^	***P<*0.001, t=-4.093**
3 days postop	197.7±45.4^*^	241.0±41.2^*^	***P<*0.001, t=-4.384**
7 days postop	78.3±35.8	81.4±35.5	*P*=0.710, t=-0.373
ESR (mm/h)			
Preop	15.5±6.1	15.9±7.2	*P*=0.825, t=-0.222
1 day postop	35.8±7.3^*^	34.7±6.9^*^	*P*=0.506, t=0.668
3 days postop	65.7±12.6^*^	67.1±13.5^*^	*P*=0.631, t=-0.483
7 days postop	43.4±9.1^*^	45.7±10.8^*^	*P*=0.326, t=-0.988
IL-6(pg/mL)			
Preop	2.8±0.7	2.9±0.8	*P*=0.556, t=-0.591
1 day postop	47.6±17.0^*^	56.3±18.4^*^	***P*=0.036, t=-2.139**
3 days postop	32.4±12.5^*^	38.6±11.7^*^	***P*=0.027, t=-2.259**
7 days postop	15.2±4.5^*^	16.6±4.9^*^	*P*=0.204, t=-1.280

CRP, C-reactive protein; CK, creatine kinase; ESR, erythrocyte sedimentation rate; IL-6, interleukin 6;

* Compared with before operation, *P* < 0.05. Significant values are in bold. Data presented as mean ± SD.

The levels of CRP, ESR, and IL-6 remained considerably elevated on day 7 after the surgery compared to before the surgery (*P*>0.05). The levels of CRP, CK, and IL-6 were significantly elevated in the MIS-TLIF group compared to the ULIF group on postoperative days 1 and 3 (*P*<0.05).

**Fig 2:**
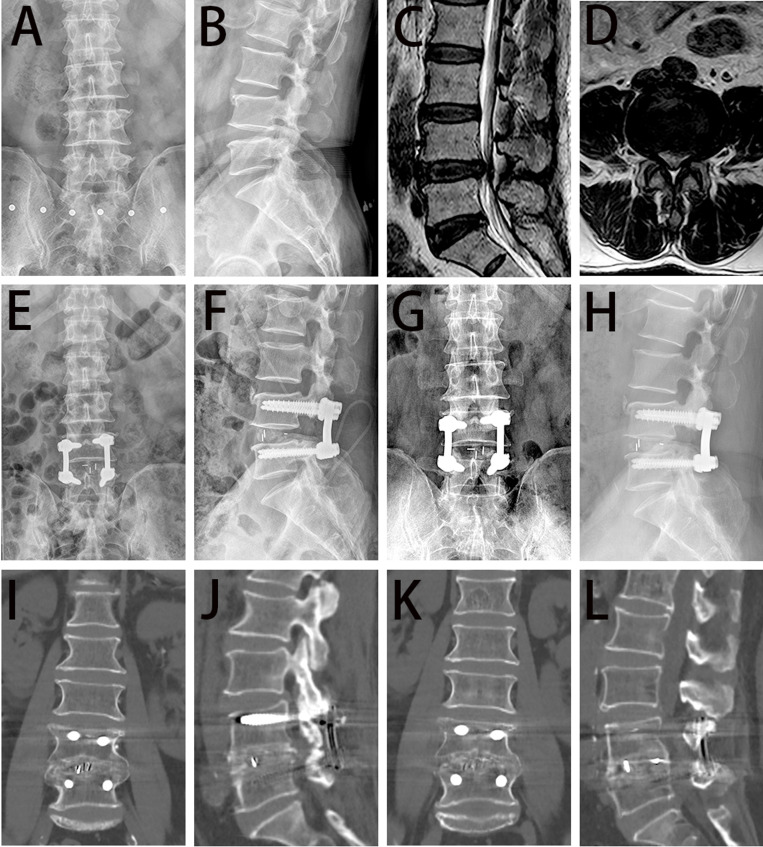
Pain and function scores in the ULIF and MIS-TLIF groups. Pre, preoperative; M, month; * *P*<0.05, ** *P*<0.01, *** *P*<0.001.

The pain and functional improvement data for both groups are presented in Fig 2, using the VAS-L, VAS-B, ODI, and JOA scores. No significant differences in preoperative VAS-L, VAS-B, ODI score, and JOA score existed across groups. The VAS-L, VAS-B, and JOA and ODI scores of both groups exhibited steady and considerable improvement over time (*P*<0.05). At one week postoperatively, the VAS-L was substantially lower in the ULIF group compared to the MIS-TLIF group (3.5±1.3 vs. 4.2±1.4, *P*=0.022). At one week and one month postoperatively, the VAS-B was considerably lower in the ULIF group compared to the MIS-TLIF group (3.9±1.4 vs. 4.6±1.5, *P*=0.029; 2.7±1.0 vs. 3.3±1.3, *P*=0.035; respectively). At one month after surgery, the ULIF group had a much lower ODI score than the MIS-TLI group (35.8±6.2 vs. 38.7±6.3, *P*=0.045). At 1 month postoperatively, the JOA score was substantially greater in the ULIF group compared to the MIS-TLIF group (19.6±2.5 vs. 18.2±2.6, *P*=0.017).

**Fig 3:**
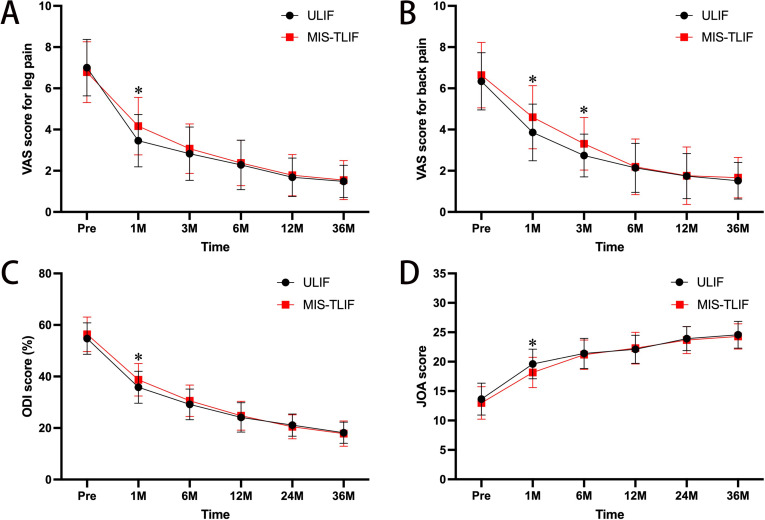
Representative imaging findings for the ULIF group are shown for a 63-year-old male. Anteroposterior (A), lateral (B) radiographs and MR (C-D) images revealed a lumbar spinal canal stenosis at the L4/5 level. Anteroposterior and lateral radiographs obtained at postoperative day 3 (E-F) and 6 months (G-H) confirm that the L4-5 internal fixation was in good position. CT (I-J) images obtained at postoperative 12 months indicate that good intervertebral fusion was achieved. CT (K-L) images obtained at postoperative 36 months indicate that good intervertebral fusion was maintained.

Regarding radiological outcomes, the fusion rate, IDH, SLA, and LLA of the two groups are shown in Table 4. The preoperative indices did not exhibit a significant difference between the two groups (*P*>0.05). At 1 year after surgery, the ULIF group had a greater interbody fusion rate than the MIS-TLIF group, although the difference did not reach statistical significance (85.7% vs. 83.3%, *P*=0.774). At 2 years and 3 years after surgery, the ULIF group had a lower interbody fusion rate than the MIS-TLIF group, although not statistically significant (91.4% vs. 92.9%, *P*=0.816; 94.3% vs. 95.2%, *P*=0.851, respectively). At 1 month, 1 year, 2 years, and 3 years after surgery, the IDH and LLA in both groups were significantly higher than those preoperatively (*P*<0.05), but no significantly difference was found between these two groups at any follow-up (*P*>0.05). At 1 month and 1 year postoperatively, the SLA in the ULIF group were significantly higher than those preoperatively (*P*<0.05). Imaging results at different time points of follow-up are shown for a representative patient in the ULIF (Fig 3, a 63-year-old male) groups.

**Table 4:** Comparison of radiological parameters between the two groups.

	ULIF	MIS-TLIF	*P* value, t/χ^2^ value
IDH (mm)			
Preop	8.7±1.2	8.4±1.1	*P*=0.152, t=1.449
1 month postop	11.0±1.1^*^	10.8±1.2^*^	*P*=0.608, t=0.515
1 year postop	10.1±1.0^*^	9.8±1.1^*^	*P*=0.231, t=1.208
2 years postop	9.7±1.2^*^	9.4±1.1^*^	*P*=0.248, t=1.166
3 years postop	9.5±1.2^*^	9.3±1.0^*^	*P*=0.400, t=0.846
SLA (°)			
Preop	8.7±2.6	8.5±3.0	*P*=0.656, t=0.447
1 month postop	9.9±2.5^*^	9.4±2.9	*P*=0.413, t=0.823
1 year postop	9.3±2.4^*^	9.1±2.9	*P*=0.813, t=0.238
2 years postop	9.1±2.2	9.0±2.9	*P*=0.909, t=0.115
3 years postop	9.0±2.3	8.9±2.8	*P*=0.941, t=0.074
LLA (°)			
Preop	45.2±6.0	43.1±4.9	*P*=0.106, t=1.635
1 month postop	50.6±5.7^*^	48.9±5.0^*^	*P*=0.173, t=1.376
1 year postop	48.8±5.7^*^	46.9±4.8^*^	*P*=0.128, t=1.538
2 years postop	47.9±5.4^*^	45.6±4.9^*^	*P*=0.056, t=1.944
3 years postop	47.1±5.3^*^	45.3±4.8^*^	*P*=0.107, t=1.630
Fusion rate (%) ^†^			
1 year postop	85.7%	83.3%	*P*=0.774, χ^2^=0.082
2 years postop	91.4%	92.9%	*P*=0.816, χ^2^=0.054
3 years postop	94.3%	95.2%	*P*=0.851, χ^2^=0.035

IDH, intervertebral disk height; SLA, Segmental lordosis angle; LLA, lumbar lordosis angle;

* Compared with before operation, *P* < 0.05. Boldface indicates statistical significance. Data presented as mean ± SD.

† According to the Bridwell interbody fusion grading criteria, grade I and II were defined as fusion.

The surgical complications are shown in Table 5. The ULIF group exhibited a decreased overall complication rate compared to the MIS-TLIF group; however, this disparity did not reach statistical significance (14.3% [5/35] vs. 16.7% [7/42], *P*=0.774). Perioperative complications in the ULIF group occurred at a rate of 8.6% (3/35), with one case of dural tear and one of transient hypoesthesia. The MIS-TLIF group had a perioperative complication rate of 9.5% (4/42), with one case of dural tear, two incidents of endplate injury, and one case of transitory hypoesthesia. All perioperative complications were cured by conservative treatment. The ULIF group had a long-term complication rate of 5.7% (2 out of 35 cases), with one instance each of symptomatic neighboring segment disease and cage subsidence diagnosed. The MIS-TLIF group had a long-term complication rate of 7.1% (3/42), including one case of symptomatic adjacent segment disease and two cases of cage subsidence. The cage subsidence was within 2 mm in both groups. As the patients were asymptomatic, they were treated conservatively. Symptomatic adjacent segment disease was significantly relieved after conservative treatment, and no surgical treatment was performed.

**Table 5:** Surgery-related complications.

Complication	ULIF (%)	MIS-TLIF (%)	*P* value, χ^2^ value
All complications	5 (14.3)	7 (16.7)	*P*=0.774, χ^2^=0.082
Perioperative	3 (8.6)	4 (9.5)	*P*=1.000, Null
Dura tear	2(5.7)	1 (2.4)	
Endplate injury	0	2 (4.8)	
Temporary dysesthesia	1(2.9)	1 (2.8)	
Long-term	2(5.7)	3 (7.1)	*P*=1.000, Null
Cage subsidence	1(2.9)	2 (4.8)	
Symptomatic ASD	1(2.9)	1 (2.4)	

ASD: adjacent segment disease; Boldface indicates statistical significance. Null: there is no statistical value when calculated using 2×2 Fisher’s Exact Test.

## Discussion

MIS-TLIF and ULIF are representative minimally invasive spinal fusion techniques introduced by Foley et al. and Heo et al., respectively, that have gradually gained wide clinical application [20–23]. MIS-TLIF is conducted using a paramedian incision, specifically targeting the interspace region between the multifidus and longissimus muscles with the aid of channel instruments and a light source. ULIF is performed through two paramedian unilateral incisions to construct endoscopic and working channels, respectively. ULIF and MIS-TLIF are both minimally invasive lumbar fusion techniques using a posterior approach. While short-term cohort studies have indicated that ULIF is associated with reduced intraoperative blood loss, postoperative pain, and a quicker recovery period in comparison to MIS-TLIF, medium and long-term follow-up has yet to validate these benefits. This study is the inaugural retrospective cohort analysis that pits ULIF and MIS-TLIF against each other in terms of mid-term clinical outcomes.

The perioperative outcomes of ULIF and MIS-TLIF were compared, revealing notable differences. ULIF exhibited a significantly longer operative time, significantly lower blood loss and postoperative drainage, as well as a shorter time to postoperative mobilization and postoperative hospital stay when compared to MIS-TLIF. The study conducted by Jiang et al. also found statistically significant differences between the two groups, with ULIF resulting in a 50-minute longer mean operative time, 70 mL less intraoperative blood loss, 60 mL less postoperative drainage, and a one-day shorter postoperative hospital stay [24]. ULIF, being a novel minimally invasive fusion method, has a learning curve, especially when the right lower extremity of the patient is involved, as the surgeon must use their left hand to operate the surgical instruments, which greatly prolongs the operative time for most right-handed surgeons. The reduction of perioperative blood loss in the ULIF group may be due to the inhibitory effect of water pressure on epidural vascular bleeding and accurate and timely hemostasis under clear intraoperative vision.

The monitoring of inflammatory factor levels helps to assess tissue damage caused by surgery as well as surgical site infection. In this study, the levels of CRP, ESR, CK, and IL-6 in both groups were significantly elevated on day 1 after the surgery, as compared to their levels before the surgery. CRP and ESR levels peaked on day three postoperatively, whereas CK and IL-6 levels peaked on day one after surgery, before declining progressively. On day seven postoperatively, CK levels returned to preoperative levels, whereas CRP, ESR, and IL-6 levels remained substantially elevated compared to preoperative levels. The ULIF group had significantly reduced levels of CRP, CK, and IL-6 compared to the MIS-TLIF group on days 1 and 3 after the surgery. The results align with the findings of Huang et al., who documented a substantial increase in CRP levels in both groups on day one after surgery, with a peak on day three. At both three and five days postoperatively, the ULIF group exhibited significantly lower CRP levels than the MIS-TLIF group. The CK value peaked on day 1 postoperatively and was considerably lower in the ULIF group compared to the MIS-TLIF group [25]. In this study, the levels of inflammatory factors in both groups showed trends of first increasing and then decreasing postoperatively, indicating no sign of infection after surgery. Nevertheless, the diminished levels of inflammatory factors in the ULIF group suggest that this surgical method inflicts less harm on the soft tissue, resulting in a milder inflammatory response.

Pain is the main complaint of patients seeking medical treatment, and rapid pain relief postoperatively is an important indicator to evaluate surgery effectiveness. In this study, both groups experienced a significant decrease in VAS-B and VAS-L scores after the surgery, compared to before the surgery. The VAS-B score shown a decrease in the ULIF group one week after the surgery, whereas the VAS-L scores showed a substantial decrease in the ULIF group at both one week and one month after the surgery. There was no significant difference in the VAS-B and VAS-L scores between the two groups at 3 months, 6 months, 12 months, and 36 months after the surgery. Similarly, Heo et al. observed that the ULIF and MIS-TLIF groups had considerably reduced VAS-B and VAS-L scores postoperatively in comparison to preoperatively, with the ULIF group having significantly reduced VAS-B and VAS-L scores on days 1 and 2 postoperatively. However, at 12 months postoperatively, VAS-B and VAS-L scores were similar between groups [26]. Thus, both surgical methods can effectively relieve patient pain. The better short-term effect of ULIF may be due to reduced damage to the paravertebral soft tissue and mild postoperative local inflammatory reaction compared to MIS-TLIF.

Enhancing functionality is crucial for patients to resume regular work and daily activities, and also significantly influences patient satisfaction. We found that both the ULIF and MIS-TLIF groups showed significant improvement in ODI and JOA scores after surgery. However, the ULIF group had a more significant improvement than the MIS-TLIF group at 1 month after surgery. Nevertheless, both groups showed equally significant functional improvement at 6 months, 1 year, 2 years, and 3 years after surgery. Similarly, Song et al. found that after surgery, both the ULIF and MIS-TLIF groups showed significantly improved ODI scores. Additionally, the ULIF group had significantly lower ODI and VAS-L scores compared to the MIS-TLIF group at 2 weeks after surgery. However, at 3 and 12 months postoperatively, the ODI scores were similar between groups [27]. Park et al. and Liu et al. found comparable results when comparing ULIF and PLIF [28,29]. Additionally, the average time to ambulation in the ULIF group was 7.2 hours shorter in the current study compared to the MIS-TLIF group. This finding is consistent with the results reported by Kim et al., wherein the ULIF group commenced postoperative activities approximately 6 hours earlier than the MIS-TLIF group [30]. Therefore, both surgical procedures are effective in improving function and early recovery is faster in ULIF. The rapid early recovery in the ULIF group can be attributed to the mild postoperative inflammatory response and pain.

Radiological findings are crucial for assessing the efficacy of fusion surgery. Frequently employed indicators comprise interbody fusion rate, IDH, SLA, and LLA.

We observed significantly improved IDH, SLA, and LLA both groups at 1 month after surgery. However, the SLA returned to the preoperative level at the 2-year follow-up, while the surgical effects of IDH and LLA persisted until 3 years after surgery. Similar findings were found by Kang et al. in a cohort trial with a 15-month follow-up period on average. In comparison to preoperative values, the IDH, SLA, and LLA in the ULIF and MIS-TLIF groups had dramatically increased at the 3-year follow-up [31]. The changes immediately after surgery may be due to the insertion of an appropriately sized interbody fusion cage and the corrective impact of fusion on the local physiological curvature. Limited by the duration of follow-up, Kang et al. did not report changes in imaging data at ≥2 years. The loss of SLA correction at 2 years postoperatively in our study may have been caused by the inconspicuous subsidence of the cage. The interbody fusion rate is the primary outcome measure used to assess the efficacy of lumbar fusion surgery. During ULIF, continuous irrigation with saline may decrease the local blood supply and osteogenesis factors, which in turn may reduce the fusion rate. However, in the present study, both ULIF and MIS-TILF groups achieved excellent interbody fusion rates. Our results are consistent with reported interbody fusion rates of 78.3%–93.3% in ULIF and 76.2–92.7% in MIS-TLIF at 1 year postoperatively [32–34]. This may be due to the ability of ULIF to prepare the bone bed meticulously and thoroughly on magnified and high-definition endoscopic images without damaging the bony endplate [35]. A 30°-spinal endoscope can be used to help thoroughly clean the contralateral cartilage endplate. However, in MIS-TLIF, the surgeon’s experience is the only factor that determines how the bone graft bed is prepared; the removal of the cartilage endplate is not always clean or the bone endplate is damaged, which increases the risk of cage subsidence and nonunion. In the future, we can take advantage of the visualization of ULIF to implant bone graft materials or fusion cages containing bone morphogenetic protein (BMP) and other bone formation-inducing substances to overcome the decreased concentration of osteogenesis factors by continuous irrigation, thereby increasing the probability of interbody fusion.

The common complications in posterior lumbar surgery include perioperative complications including dural tear, nerve injury, and endplate injury, and long-term postoperative complications including cage subsidence and adjacent segment disease. Reducing the incidence of complications can accelerate postoperative recovery and improve quality of life. In their 13-month cohort trial, Heo et al. found a lower overall complication rate in the ULIF group than in the MIS-TLIF group (8.7% vs. 13.0%), but the difference was not significant [36]. In this study, the ULIF group had a slightly reduced overall complication rate compared to the MIS-TLIF group (14.3% vs. 16.7%), although this difference was not statistically significant. The complication rate in both groups was higher than that reported by Heo et al., likely due to the longer follow-up period in our study. It is well known that the incidence of symptomatic adjacent segment disease (ASD) increases gradually with the extension of follow-up time. In the present study, one patient in the MIS-TLIF group developed symptomatic ASD at 1 year after surgery, and one patient in the MIDLIF group at 2 years after surgery, which was the main reason for the higher complication rate in this study. Furthermore, complications of posterior lumbar fusion are independently associated with multiple surgical levels (>2), extended operation duration, and increased intraoperative blood loss [37,38]. The complication rates in our study showed no statistically significant difference between the two groups. This may be attributed to the lower perioperative blood loss in the ULIF group, despite the significantly longer operative time.

There are certain constraints in the present study. Firstly, this investigation was conducted as a retrospective cohort study rather than a prospective randomized controlled study. Despite the statistical processing of the data, bias is inevitable, which may affect the conclusions. Second, the small sample size increased type II errors and reduced our ability to detect small events and confidently identify actual differences in the incidence of close events between the groups. Furthermore, varying proficiency between surgeons also contributes to a bias in conclusions. Consequently, prospective, randomized, controlled studies with large samples are required to further determine the benefits of ULIF surgery.

In conclusion, ULIF and MIS-TLIF achieved equally excellent results in interbody fusion rate, IDH, SLA, LLA, complication rate, long-term pain relief, and functional improvement in single-level LDD. However, ULIF had obvious advantages in perioperative blood loss, early postoperative pain relief and functional improvement, time to ambulation as well as postoperative hospital stay, although its current shortcomings include long operation time and relatively high technical requirements.

## Supporting information

S1 File Demographic information, Blood loss, Inflammatory factors, VAS scores, Function scores, Complications and Imaging parameters. (ZIP)

S2 File blood loss. (ZIP)

S3 File Inflammatory factors. (ZIP)

S4 File VAS scores. (ZIP)

S5 File Function scores. (ZIP)

S6 File complications.xlsx. (ZIP)

S7 File Imaging parameters. (ZIP)
